# Development of Composite Aluminosilicate Materials Based on Iron–Carbon Fly Ash from CHPP-2: A Comparative Analysis of the Effect of Saryozek and Alekseevskaya Clay Structural Types on Phase Formation During Semi-Dry Pressing

**DOI:** 10.3390/molecules31162799

**Published:** 2026-08-11

**Authors:** Gulnaz Adilbayeva, Sestager Aknazarov, Olga Golovchenko, Aigul Abisheva, Zhanibek Amir, Makhmud Biisenbayev, Ainur Muratova, Assem Zh. Askarova, Aitugan Sabitov

**Affiliations:** 1 Department of Chemical Physics and Materials Science, Al-Farabi Kazakh National University, Al-Farabi Avenue 71, Almaty 050040, Kazakhstan; adilbaevagulnaz@mail.ru (G.A.); sestager@mail.ru (S.A.); teya290965@gmail.com (O.G.); 2 Scientific and Technical Center “Zhalyn”, Pavlodarskaya Street 11, Almaty 050050, Kazakhstan; bijsenbay@gmail.com; 3 Faculty of Natural Sciences, Kazakh National Women’s Teacher Training University, Gogol Str., 114, k. 1, Almaty 050000, Kazakhstan; abisheva.ak@mail.ru (A.A.); amirjanibek@mail.ru (Z.A.); 4 Alfa-Standard Center for Expertise and Certification, Seifullin Avenue 531, Almaty 050000, Kazakhstan; ainur.m7k@mail.ru (A.M.); assem.askarova88@gmail.com (A.Z.A.)

**Keywords:** composite materials, Fe-Al-C-Si system, fly ash, montmorillonite clay, kaolinite, semi-dry pressing, phase formation, TG/DTA, compressive strength

## Abstract

This study presents a comparative analysis of the effect of the structural–mineralogical type of clay matrices on the phase and structure formation in composite aluminosilicate materials within the multi-component Fe-Al-C-Si system. Highly plastic Saryozek montmorillonite clay and moderately plastic Alekseevskaya kaolinite–illite clay were investigated as binding matrices to consolidate iron–aluminosilicate fly ash from the Almaty CHPP-2. The raw materials and binary batches containing 10 to 50 wt.% fly ash were evaluated using XRD, XRF, TG/DTA, and SEM techniques. The results demonstrate that the superior plastic and binding properties of the Saryozek clay ensure enhanced consolidation of the non-plastic, fragmented ash particles. Simultaneous thermal analysis reveals that increasing the compaction pressure from 20 to 30 MPa induces a kinetic shift in the montmorillonite dehydroxylation interval toward higher temperatures (580–720 °C) due to increased partial water vapor pressure within the dense green body. This thermal shift scientifically necessitates introducing an isothermal dwell at 600 °C to mitigate firing defects. The optimal composite properties are achieved at a molding pressure of 30 MPa, a firing temperature of 1050 °C, and a fly ash concentration of 10–20 wt.%, yielding a peak compressive strength of 38.4 MPa. SEM confirmed that under these conditions, the locally formed silicate melt uniformly encapsulates the crystalline mullite and quartz microparticles, whereas increasing the ash content to 50 wt.% results in a loose, highly porous structure that degrades strength down to 17.9 MPa. These findings lay a scientifically substantiated foundation for optimizing composite ceramic synthesis and reducing structural defects.

## 1. Introduction

The development of modern materials science is closely linked to the elaboration of scientific foundations for designing multi-component composite systems with a tailored set of performance characteristics. Among the promising research trends, a special place is occupied by the engineering of composites based on the quaternary Fe-Al-C-Si system. Interest in objects with this elemental profile is driven by the possibility of forming unique phase conglomerates that combine rigid crystalline frameworks (mullite, sillimanite, carbide, or silicide phases) with plastic matrices of various physicochemical natures [1,2].

Traditionally, research on alloys and cermet composites within the Fe-Al-C-Si system has focused on enhancing their mechanical and thermal characteristics. However, from the standpoint of an interdisciplinary materials science approach and the principles of green chemistry, there is fundamental interest in discovering affordable, natural-technogenic sources of these elements. From this perspective, ash and slag waste from thermal power plants—specifically the fly ash generated from the combustion of high-ash Ekibastuz coal—represents a unique object for investigating phase formation processes within the Fe-Al-C-Si system [3,4].

This disperse product is characterized by the stable presence of all four base elements: silicon and aluminum (in the form of glass phase, quartz, and refractory mullite), iron (as isomorphic impurities and Fe_2_O_3_), and carbon (in the form of residual unburned coke). Such a chemical–mineralogical profile allows fly ash to be considered not merely as an inert filler, but as a functional iron–aluminosilicate component with an embedded carbonaceous reducing agent, capable of actively participating in high-temperature sintering processes and phase transformations when interacting with plastic matrices [5,6,7]. To better understand these complex interactions, the fly ash generated from the combustion of high-ash Ekibastuz coal at Almaty CHPP-2 is selected as a strategic object of study. Its quaternary elemental profile combines rigid refractory frameworks (mullite and quartz) with a notable proportion (~5 wt.%) of residual unburned carbon. While the presence of this carbonaceous fraction introduces technological challenges—such as increased moisture demand and the necessity for a controlled low-temperature burnout stage to prevent structural bloating—its exact influence on phase formation and pore structure under semi-dry pressing requires systematic investigation.

A number of fundamental domestic and foreign studies have been dedicated to the use of ash and slag waste in the composition of composite and building materials. In a large-scale review by M. Kuźnia, the environmental, energy, and materials science aspects of the utilization of fly ash generated from coal combustion are examined in detail [8]. The author emphasizes that in the context of a circular economy, fly ash rich in SiO_2_, Al_2_O_3_, and Fe_2_O_3_ oxides represents a valuable anthropogenic raw material suitable for the modification of polymer, cement, and silicate systems. Similar conclusions are drawn in the work of X. Lu et al., which synthesizes global experience in the large-scale recycling of coal ash. The study of phase formation processes during the integration of waste into aluminosilicate binders demonstrates that the thermal consolidation of components enables the effective encapsulation of heavy metals within the crystalline matrix, thereby addressing concomitant environmental challenges [9].

The applied aspects of fly ash utilization in related hydraulic binder systems were investigated by R. Mohanraj. Using composite concretes as an example, the authors established that the introduction of ultradispersed silica (nano- SiO_2_) in controlled dosages accelerates hydration and stimulates the formation of calcium silicate hydrate (C-S-H) gel, which promotes the forced compaction of the structure and reduces overall porosity. Conversely, an excess of the disperse additive provokes undesirable particle aggregation and a localized loss of strength [10].

The specific features of high-temperature behavior and phase transformations in composites based on clay raw materials and fly ash are described in a study by E. Plevová. Using smectite group minerals (montmorillonite) as an example, the authors established via simultaneous thermal analysis that the introduction of fly ash shifts the temperature intervals of dehydration and dehydroxylation, while the total mass losses upon heating strictly correlate with the crystallochemical characteristics and defectiveness of the initial clay matrix [11]. Trends in the co-utilization of fly ash and complex industrial wastes, such as electrolytic manganese residue, convincingly demonstrate the close relationship between firing parameters, porosity, and the fixation of the final phase composition, where additives of variable-valence oxides can act as sintering mineralizers [12].

The experimental results of E. Fidancevska et al. prove that fly ash can effectively replace up to 40% of natural clay in the production of dense composites, while the kinetics of compaction and sintering are limited by liquid phase formation within the temperature range of 1050–1100 °C [13]. Particle geometry plays an important role in regulating the internal topology of composites. The use of narrow ash fractions—hollow silicate microspheres (cenospheres)—allows for a reduction in the average density of products by ~36% while simultaneously increasing dynamic impact fracture toughness, which is critically important for roofing materials [14]. Similar effects were recorded by K. Rugele et al. during the synthesis of lightweight syntactic aluminosilicate foams with a clay binder [15]. From a micromechanical standpoint, the porous structure of the composites was characterized in detail in the work of L.F. Dutra et al., where, based on textural and thermal analysis, it was shown how the destruction of hydrosilicates during firing predetermines the geometry of the resulting pores and capillaries [16]. Finally, investigations into the dynamics of elastic properties under polythermal conditions revealed that replacing the traditional non-plastic (lean) component with fly ash in amounts up to 40 wt.% significantly alters the nature of the green body’s thermal expansion. The authors proved that during the cooling of the fired material, a critical role is played by internal tensile stresses arising around the grains of residual quartz during the polymorphic beta-to-alpha transition, which initiates local microcracking and directly predetermines the final Young’s modulus of the composite [17].

In most of the known technological schemes, fly ash is used as a passive thinning or porizing component. However, when switching to the semi-dry pressing method, an increase in the ash concentration in the masses often leads to a sharp decrease in the strength characteristics of the pressing and the finished composite. This is due to the non-plastic nature of the ash particles, their angular fragmentation geometry, as well as the presence of residual carbon, which prevents early consolidation. To stabilize the properties of a multicomponent system, precision selection of an optimal natural clay matrix with high binding and plastic potential is required.

In the scientific and technical literature, the issues of the comparative influence of the structural and mineralogical types of the clay crystal lattice on the processes of phase formation of ferruginous–carbonaceous fly ash under conditions of increased molding pressures are not fully covered. The behavior of the labile crystal lattice of minerals of the montmorillonite group (with layers capable of expansion) and the more rigid, stable lattice of kaolinite–illite clays under conditions of synchronous pressure and temperature action differs significantly [18]. This has a direct effect on the kinetics of dehydroxylation, the amount of partial vapor pressure inside the pores, the viscosity of the resulting silicate melt and, as a result, on the porous structure and strength characteristics of composites.

From a mechanics and geotechnical perspective, the effectiveness of semi-dry pressing and structural consolidation is fundamentally governed by compaction dynamics, interparticle contact mechanics, and physicochemical reactions within the composite matrix. Applied compaction pressure forces the spatial convergence of dispersed particles, reducing interparticle voids and optimizing the interfacial contact between the plastic clay matrix and non-plastic mineral frameworks. Furthermore, as confirmed by advanced microstructural and finite element investigations, high compaction forces directly influence the internal stress distribution and restrict the escape kinetics of volatiles, making the precise adjustment of forming pressure and high-temperature thermal regimes crucial for mitigating structural defects, enhancing load transfer, and maximizing the compressive strength and deformation resistance of consolidated aluminosilicate composites [19,20].

The purpose of this work is a comparative study of the influence of the structural and mineralogical type of clays of the Saryozek (montmorillonite type) and Alekseevskaya (kaolinite-illite type) deposits on the processes of phase formation, the kinetics of thermal behavior, and the physico-mechanical properties of composite aluminosilicate materials based on ferrous–carbon ash from the CHPP-2 in Almaty when the parameters of semi-dry pressing are varied. To achieve this goal, the following tasks were solved:

- To study the chemical and mineralogical, granulometric composition and rheological parameters of the initial clays of Saryozek and Alekseevskaya, as well as the fly ash of CHPP-2 in Almaty.
- To investigate the effect of the specific pressure of semi-dry pressing (20–30 MPa) on the high-temperature behavior, mass change and temperature ranges of dehydroxylation of the developed binary charges by synchronous thermal analysis (TG/DTA) methods.
- To evaluate the effect of the ash component concentration (10–50 wt.%) and the firing temperature (950–1050 °C) on the complex of physical and mechanical properties (compressive strength, water absorption, fire shrinkage) of synthesized composite materials.
- To carry out a scanning electron microscopic analysis (SEM) of the chipped samples to determine the nature of the microstructure, phase distribution and porosity, depending on the degree of filling of the silicate matrix with the ash component.

## 2. Results and Discussion

### 2.1. Granulometric Composition and Chemical Characteristics of Ash and Clay Samples

The granulometric composition is one of the main characteristics determining the rheological properties of the initial components for the preparation of ceramic materials, as well as the sorption abilities of the finished products. Structure formation during firing and plasticity also play a key role. The higher the content of microdispersed particles, the higher the plasticity of the material, which ensures a strong bond and positively affects the strength characteristics of the final products.

Results of analysis of the granulometric composition of ash and clay samples are shown in Table 1. Sampling and sample preparation of raw materials for analysis were carried out according to the generally accepted ASTM methodology [21].

Saryozek clay has the most highly dispersed structure, in which the content of a fine fraction less than 0.056 mm in size reaches a maximum value of 46.2%, which determines its high plasticity and binding ability. In turn, Alekseevskaya clay is characterized by a predominance of the average fraction (49.4% on a 0.056 mm sieve) with a minimum number of large inclusions (12.5%). Unlike raw clays, the CHPP-2 ash sample shows significant heterogeneity with a high proportion of coarse-grained fraction (34.2% on a 0.63 mm sieve) due to the presence of aggregates and products of underburning. The differentiated data obtained make it possible to compare the high dispersion of individual Saryozek clay with the enlarged granulometric profile of CHPP-2 ash, characterized by an increased proportion of coarse-grained fraction.

**Figure 1:**
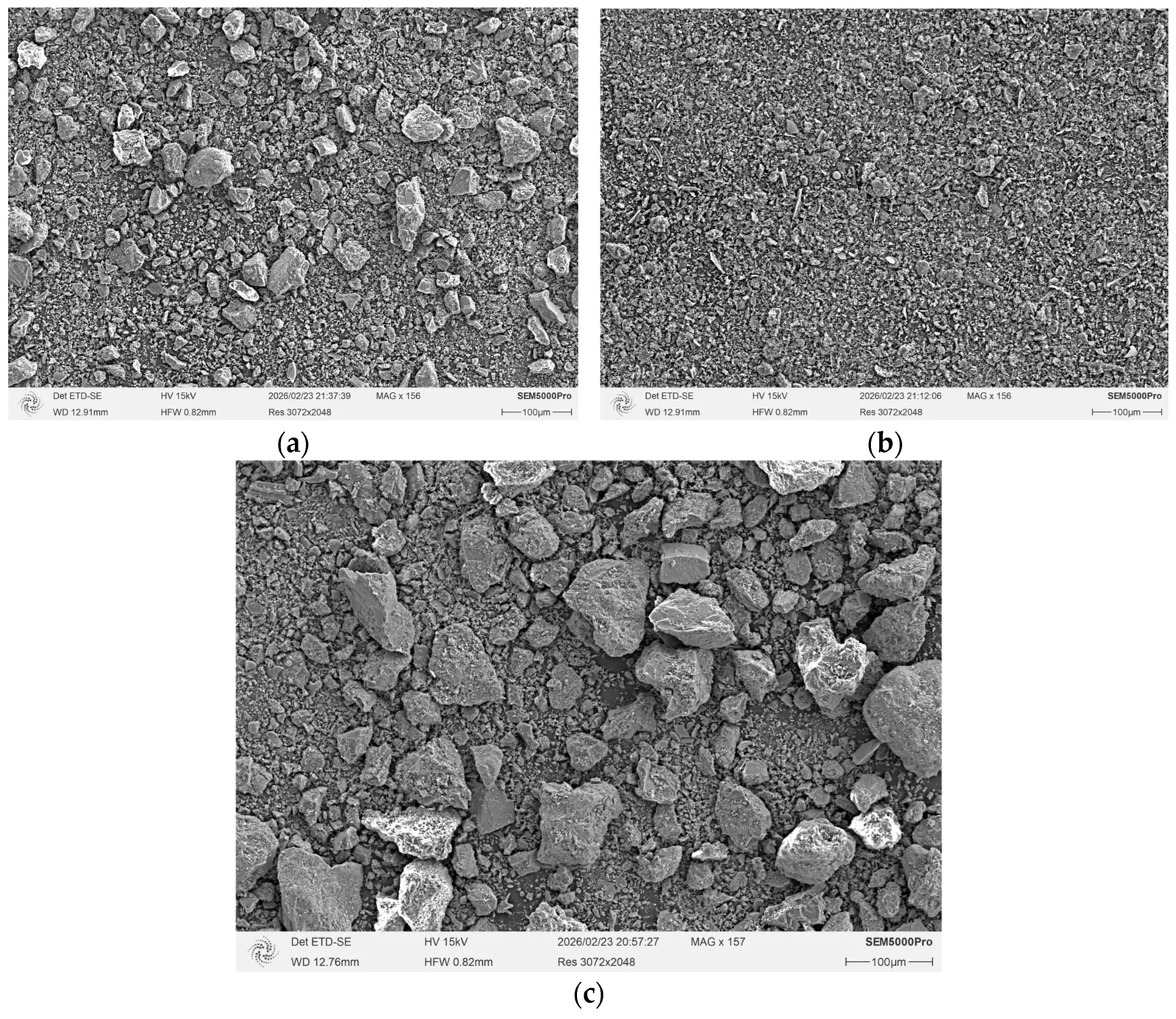
SEM micrographs of the initial raw materials: (**a**) Saryozek clay; (**b**) Alekseevskaya clay; (**c**) CHPP-2 fly ash.

To visualize in detail the shape of the particles, the nature of their spatial organization, and the verification of the sieve analysis data, the initial components were studied using SEM (Figure 1).

Micromorphological analysis of the Saryozek clay sample (Figure 1a) indicates the presence of large, irregularly shaped aggregated clusters surrounded by a dense array of highly dispersed microparticles. This pattern confirms the mixed profile of granulometry. On the contrary, the microstructure of Alekseevskaya clay (Figure 1b) is represented by a more homogeneous, ordered lamellar-layered (scaly) texture, where the particles are combined into plane-parallel packages [22].

An electron microscopic image of the CHPP-2 fly ash (Figure 1c) demonstrates a fundamentally different, rigid fragmentation geometry. The man-made component consists of randomly distributed, sintered aggregates of irregular angular shape with sharp edges, which, as part of the ceramic charge, will serve as a non-plastic frame filler.

**Figure 2:**
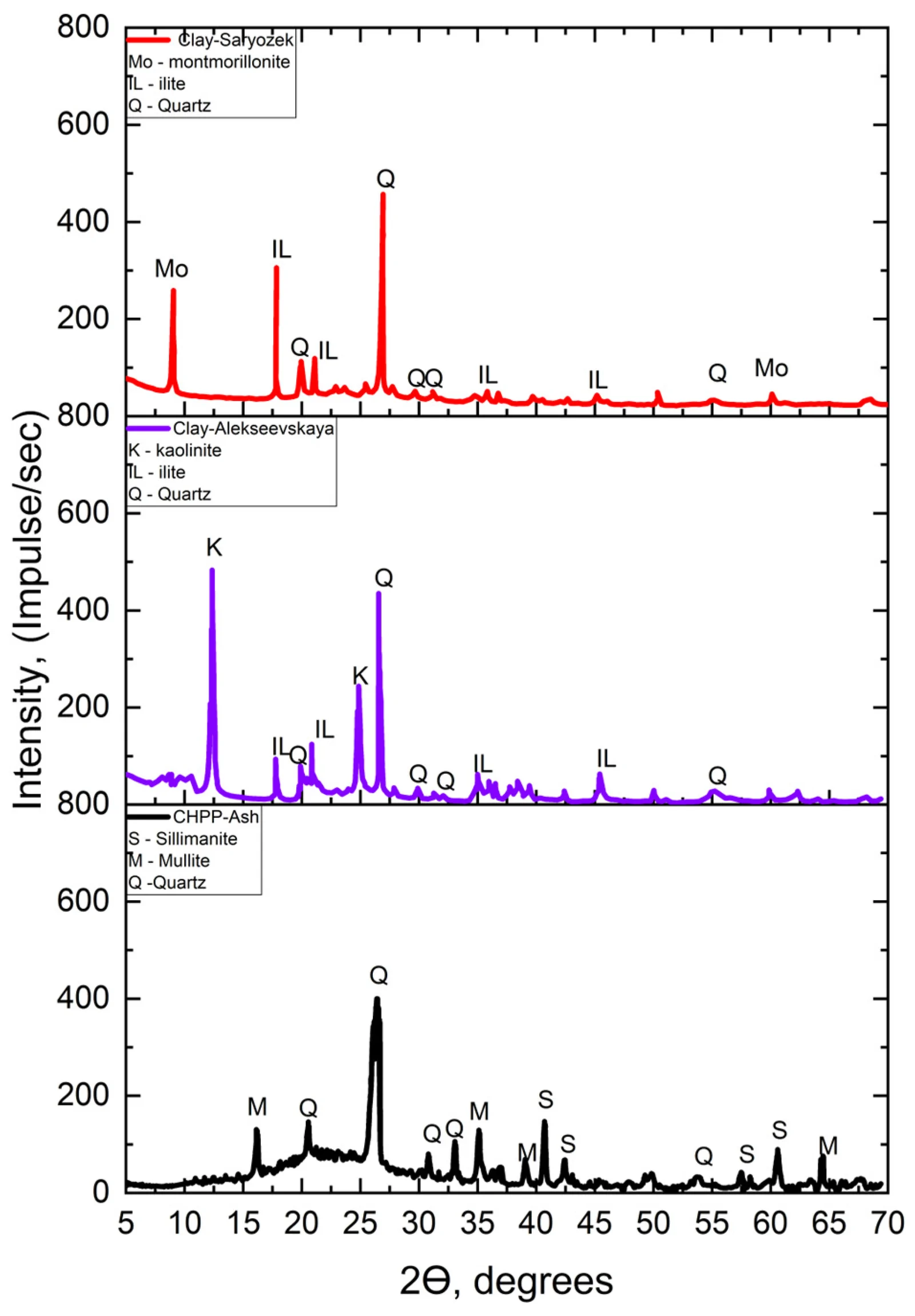
Diffractograms of the studied raw material samples: red color—clay of the Saryozek deposit (montmorillonite type); purple color—clay of the Alekseevskaya deposit (kaolinite-illite type); black color—fly ash of the CHPP-2 Almaty (ferruginous-aluminosilicate filler).

The chemical and phase composition of raw materials is a determining factor in the production of ceramic products. A DRON-4 diffractometer (STC Burevestnik, St. Petersburg, Russia) using the powder diffractogram method was used for the analysis. XRD allows to determine the phase composition of the formations and their percentage. Figure 2 shows the diffractograms of the studied samples.

A comparison of the phase and granulometric compositions of the studied materials demonstrates a direct relationship between their crystallochemical nature and dispersed characteristics. Thus, the most highly dispersed structure of Saryozek clay, containing 46.2% of a fraction less than 0.056 mm, is due to the predominance of montmorillonite (Mo) in its composition, a mineral with a labile crystal lattice prone to dispersion into the thinnest flakes. On the contrary, the thermodynamically more stable kaolinite (K) dominates in Alekseevskaya clay, crystallizing in the form of large and dense packages, which is consistent with a high proportion of the average fraction (49.4% on a 0.056 mm sieve) and its smaller specific surface area. Unlike hydrophilic clay, the sample of technogenic ash of CHPP-2 is characterized by pronounced heterogeneity and an increased content of coarse-grained fractions (34.2% on a 0.63 mm sieve). A similar granulometric profile of fly ash is confirmed by its diffraction pattern, where peaks of hard, sintered high—temperature phases—mullite (M) and sillimanite (S), as well as free quartz (Q)—are recorded. The revealed structural and mineralogical correlations show that the highly dispersed montmorillonite matrix of Saryozek clay has pronounced hydrophilic properties, while the individual technogenic ash of CHPP-2 is a non-plastic aluminosilicate component with a rigid crystalline framework.

The chemical composition of the studied samples of two types of clay and ash of CHPP-2 is shown in Table 2.

The chemical analysis data presented in Table 2 are in full agreement with the results of X-ray phase and granulometric analyses of the studied materials. The high content of aluminum oxide (24.02%) in Alekseevskaya clay objectively confirms its kaolinitic nature, which determines its predominantly medium-fractional composition due to the rigid packages of the crystal lattice. On the contrary, the increased concentration of SiO_2_ (64.78%) in the montmorillonite clay of Saryozek indicates a significant amount of free fine quartz, intensifying its grinding to fine fractions of less than 0.056 mm. The presence of a significant amount of alkaline oxides K_2_O (3.5%) and Na_2_O (2.08%) in Alekseevskaya clay indicates the presence of hydrous minerals (illite) and determines the high sinterability of the charge due to the formation of low-melting eutectic. Oxide profile of CHPP-2 ash (SiO_2_ = 58.8%; Al_2_O_3_ = 21.26%) explains the mechanism of high-temperature synthesis of the crystalline phases of mullite and sillimanite recorded on the diffraction pattern. The increased content of iron oxide (6.6%) in combination with the residual carbon of underburning (5.0%) determines the specifics of the chemical composition of CHPP-2 ash as a ferruginous alumosilicate man-made raw material with an individual reserve of organic component. R_2_O represents the total content of oxides of minor alkali metals, which can be Li, Cs. According to the analysis, the ash is represented by the following minerals: SiO_2_—quartz, mullite—Al_1.272_Si_0.798_O_4.864_ (Al_6_Si_2_O_13_—Al_4_SiO_8_) where the composition is not constant, sillimanite—Al_2_SiO_5_, calcite- CaCO_3_, rutile—TiO_2_, and magnesite—MgCO_3_.

Table 3, Table 4 and Table 5 show the data on the phase composition of the raw material based on the analysis results.

The results of the quantitative phase analysis of the independently studied raw material components, presented in Table 3, Table 4 and Table 5, revealed mineralogical differences between Saryozek and Alekseevskaya clays, as well as CHPP-2 ash. Alekseevskaya clay belongs to the kaolinite type (43.2% kaolinite) with an illite hydrosilicate content of 15.1%, which determines the high thermal stability of its individual crystalline matrix. Saryozek clay is a montmorillonite raw material (45.4% montmorillonite), the labile crystal lattice of which determines the hydrophilic properties of this material.

The initial ash of CHPP-2 is characterized by a predominance of a rigid mullite frame (45.6%) and crystalline quartz (36.3%), which makes it possible to classify it as a high-temperature nonplastic component. The presence of glass phase (4.6%) and albite (5.8%) in the composition of individual ash, as well as feldspar in Saryozek (7.8%) and Alekseevskaya (2.8%) clays indicates their different potential for liquid-phase sintering when heated. The decarbonization of calcite (3.5%) and magnesite (0.9%) in the ash residue, as well as the dehydration of gypsum (3.2–3.5%) in both clays, record the temperature ranges of gas release for each isolated component.

### 2.2. Physical and Mechanical Properties of the Starting Materials

According to the standard methodology, the parameters of the starting materials were evaluated—density and physico-mechanical characteristics (compressive strength, water absorption, fire shrinkage, etc.) [23,24].

The studied plasticity of clay samples is shown in Table 6.

The results of determining the plasticity characteristics of the investigated clay raw materials, presented in Table 6, are in strict correlation with their quantitative phase composition. The high values of the liquid limit (59.03%) and plastic limit (35.82%) of Saryozek clay are attributed to intensive coagulation structure formation and the well-developed hydrophilic shell of the montmorillonite phase. Consequently, the calculated plasticity index of the Saryozek sample reaches 23.21%, which allows this raw material to be classified as highly plastic, possessing a significant binding capacity with respect to non-plastic ash and slag waste. Conversely, substantially lower boundaries of the plastic state were recorded for the kaolinite clay from Alekseevskaya (the plastic limit is 21.45%), which is explained by the rigid two-layer crystal lattice of kaolinite, which binds water only via the outer surface of its packets. The smaller molding moisture interval of the Alekseevskaya deposit is confirmed by a plasticity index at the level of 14.40%, positioning it as a moderately plastic stabilizing component.

The physical and mechanical characteristics of the investigated clay within the temperature range of 950–1200 °C, used to determine the refractory properties of the clays employed, are presented in Table 7.

A comparative analysis of the physical and mechanical testing data revealed a dependence of the high-temperature sintering kinetics of the samples on their mineralogical and particle size compositions. The kaolinitic Alekseevskaya clay, containing 43.2% kaolinite, is characterized by a wide sintering interval; with a temperature increase from 950 to 1200 °C, the water absorption of the samples decreases from 15.9% to 4.5%, while the compressive strength monotonically increases from 16.8 to 35.1 MPa. The total shrinkage of this material increases from 3.15% at 950 °C to a maximum of 8.1% at 1150 °C, followed by a decrease to 6.7% at 1200 °C without loss of structural integrity.

The montmorillonitic Saryozek clay, containing 45.4% montmorillonite, begins to actively densify in the range of 950–1050 °C, where the water absorption drops from 18.2% to 9.8% and the strength increases from 12.4 to 25.3 MPa. The maximum strength for this sample is recorded at 1100 °C, reaching 28.9 MPa at a total shrinkage of 10.12% and a water absorption of 6.2%. Further heating to 1150 °C leads to pyroplastic softening and bloating of the ceramic body, which is manifested by a drop in strength to 24.1 MPa and a decrease in total shrinkage to 9.40%, while at 1200 °C, complete deformation of the samples occurs. The obtained results demonstrate the thermal stability limitations of pure montmorillonitic Saryozek clay at temperatures above 1100 °C, which necessitates finding pathways for its high-temperature stabilization. The individual physical, mechanical, and thermal properties of the CHPP-2 fly ash component are presented in Table 8.

The data on the physical and mechanical characteristics of the initial CHPP-2 fly ash, presented in Table 8, determine its individual structural and thermal parameters. The bulk density of the disperse waste in its loose state is ~900 kg/m^3^, with a true grain density of 1.98 g/cm^3^. The fineness of the ash component is characterized by a specific surface area of 260–280 m^2^/kg. The high individual thermal constants—a softening point at 1300 °C and a melting temperature at 1430 °C—are thermodynamically governed by the dominance of refractory mullite (45.6%) and crystalline quartz (36.3%) in the phase composition of the ash. These indicators confirm the high refractoriness of the isolated ash residue.

An important stage toward the utilization of raw materials for structural ceramics is their classification, which is based on the most characteristic quality criteria of the material used [25]: basicity modulus (hydraulic modulus), BM; acidity modulus, AM; silicate modulus, SM; and quality coefficient (hydraulic activity) (Table 9).

An analysis of the calculated classification parameters presented in the summary Table 9 allows for a quantitative evaluation of the phase formation nature and the reactivity of the investigated aluminosilicate systems. The low basicity modulus values for all components determine their classification as an acidic type of raw material, with a sharp predominance of glass-forming silicon and aluminum oxides over alkaline earth fluxes. The acidity modulus and silicate modulus sequentially increase in the series: Alekseevskaya clay→CHPP-2 fly ash→Saryozek clay, which directly correlates with the increasing fraction of free silica in the structure of each individual material. The quality coefficient (Qc) values recorded in the range of 0.345–0.407 classify the investigated fly ash and clays as autonomous, inert, low-calcium aluminosilicate materials.

Based on a comparative analysis of the properties of the two investigated clays, the clay from the Saryozek deposit was selected as the base aluminosilicate raw material for the synthesis of structural ceramics. In contrast to the moderately plastic kaolinitic Alekseevskaya clay, the high fineness and dominance of the montmorillonite phase in the Saryozek clay provide it with enhanced plasticity and binding capacity. This rheological potential is critically required to compensate for the leaning effect of the non-plastic industrial waste—CHPP-2 fly ash—to ensure a uniform distribution of the components within the molding mixture, and to obtain high-quality green bodies via semi-dry pressing. The drawback identified during thermal testing of the pure Saryozek clay—namely, a narrow sintering interval followed by pyroplastic softening and deformation at temperatures above 1100 °C, whereas the Alekseevskaya clay maintains structural stability up to 1200 °C—is completely mitigated by the introduction of the ash residue. The high individual softening and melting temperatures of the fly ash, governed by its mullite–quartz composition, form a rigid, thermally stable framework that prevents destructive bloating and shrinkage of the montmorillonite matrix during high-temperature firing.

### 2.3. Optimization of Technological Parameters for Composite Ceramic Fabrication

The batch mixtures for ceramic preparation were calculated with varying fly ash content added to the clay in wt.%: 10, 20, 30 and 50. The chemical composition of the batches was calculated based on the XRF data of the initial materials, as presented in Table 10.

Based on the analytical data of the raw materials used, investigations were conducted to determine the optimal technological parameters for producing structural ceramics with physical and mechanical characteristics meeting the specified requirements.

Table 11 and Table 12 present the effects of molding pressure, batch composition, and firing temperature on the physicochemical indicators of the ceramics at 20 MPa.

A comparative analysis of the experimental data (Table 11 and Table 12) allows for the establishment of complex physicochemical regularities in the sintering of the binary “Saryozek clay—CHPP-2 fly ash” systems, depending on the processing parameters of molding and heat treatment. Altering the molding pressure from 20 MPa to 30 MPa exerts a differentiated effect on the structurally sensitive properties of the samples.

Thus, the firing shrinkage value in all investigated formulations demonstrates invariance to changes in the molding pressure, recording identical values at both 20 MPa and 30 MPa (for instance, a stable decrease in shrinkage down to 1.9% at the maximum fly ash loading of 50%). This proves that the rigid mullite–quartz framework of the fly ash, acting as a non-deformable leaning filler, is the decisive factor in the spatial stabilization of the montmorillonite matrix, regardless of the initial compaction degree of the batch [15,19].

Increasing the molding pressure to 30 MPa leads to an expected and significant decrease in the water absorption of the ash–clay composites across the entire temperature range. For the formulation containing 10% ash at a firing temperature of 1050 °C, the water absorption decreases from 18.3% (at 20 MPa) to 14.92% (at 30 MPa). This effect is attributed to forced capillary contraction: a higher mechanical compaction force brings the angular, clastic particles of the industrial waste and the flaky aggregates of montmorillonite closer together, minimizing the volume of interparticle voids and compensating for the porosity arising from the burnout of 5.0% carbonaceous unburned residue in the ash [26].

The effect of molding pressure on the compressive strength exhibits a contrasting behavior for the ash-free and ash–clay systems. In the control samples fired at 1050 °C, increasing the pressure to 30 MPa causes a decrease in strength down to 32 MPa, indicating over-compaction of the pure Saryozek clay, which leads to structural destruction during dehydration. The decrease in compressive strength observed for the pure Saryozek clay samples when the molding pressure is increased to 30 MPa is attributed to high internal shrinkage stresses and microstructural defects. As шe established by thermal analysis (see next Section 2.4), the dense packing at 30 MPa significantly restricts the escape kinetics of structural volatiles. In pure, highly plastic montmorillonite, this high compaction combined with pronounced firing shrinkage (reaching over 10% as shown in Table 7) generates severe internal thermal-shrinkage stresses and microcracking. Conversely, introducing the rigid mullite–quartz framework of the fly ash creates a porous, dimensionally stable skeleton that mitigates these internal stresses and prevents structural degradation.

Conversely, in the composite formulations, increasing the pressure to 30 MPa provides a sharp, synergistic growth in strength: for the samples containing 10% CHPP-2 fly ash, the strength increases from 23.9 MPa to an extreme value of 38.4 MPa, and for the 20% formulation, it rises from 21.4 MPa to 26.3 MPa.

The physicochemical mechanism of the recorded optimum (10–20% CHPP-2 fly ash, 30 MPa, 1050 °C) lies in the fact that the elevated pressure ensures a maximum interfacial contact area between the clay and the ash. At a temperature of 1050 °C, the locally softened, highly disperse montmorillonite matrix, in combination with low-melting eutectics, effectively encapsulates and cements the densely packed, framework microparticles of mullite and quartz, thereby forming a monolithic composite structure [27]. When the ash concentration is increased further to 30–50%, the strength decreases under both pressure regimes due to a pronounced deficit of the plastic binding phase.

Limiting the maximum heat treatment temperature to 1050 °C is dictated by the physicochemical destructive processes that accelerate within the montmorillonite matrix upon further heating. Elevating the firing temperature to 1100 °C and above leads to a sharp decrease in the viscosity of the resulting silicate melt and drives the system into a state of pyroplastic softening, which is clearly confirmed by the deformation of the pure Saryozek clay at 1200 °C (see Table 7). The introduction of the rigid mullite–quartz framework of the CHPP-2 fly ash partially compensates for this drawback at 1050 °C; however, at temperatures exceeding this threshold, a process of destructive gas bloating is initiated within the bulk of the composite. This, in turn, leads to a significant loss in the strength of the product [28,29].

### 2.4. Thermal Analysis of Phase Transformations in Saryozek Montmorillonite Clay and Fly Ash

**Figure 3:**
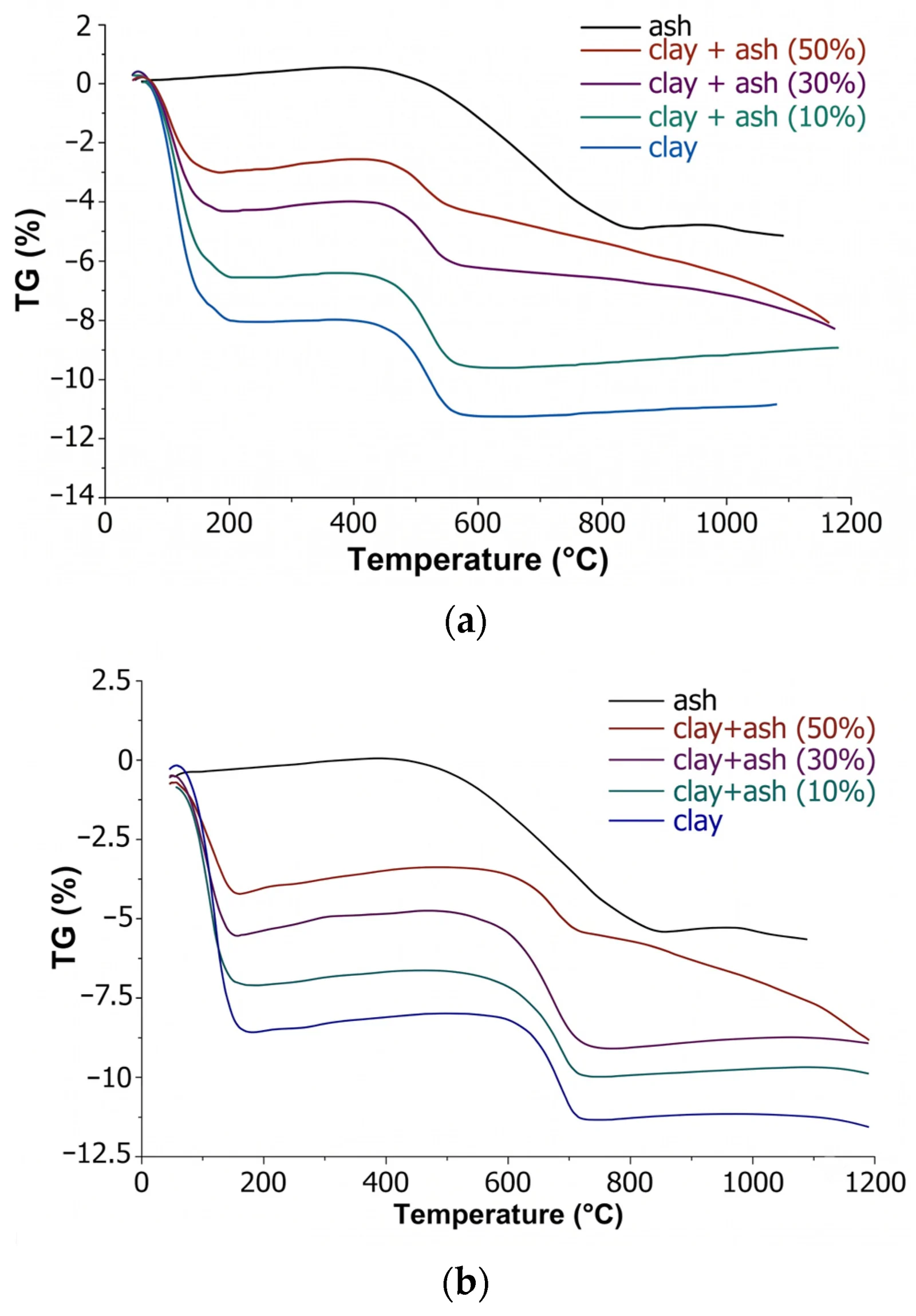
Thermogravimetric (TG) curves of the initial components and ash–clay compositions obtained at various compaction pressures: (**a**) 20 MPa, (**b**) 30 MPa.

To establish the physicochemical and thermodynamic regularities of phase transformations during the firing of the developed materials, a comparative thermogravimetric analysis (TG) was performed. Figure 3 presents the mass loss curves of the individual components (pure Saryozek clay and CHPP-2 fly ash) and the binary ash–clay compositions with industrial filler contents of 10%, 30%, and 50%, molded at compaction pressures of 20 MPa (Figure 3a) and 30 MPa (Figure 3b).

**Figure 4:**
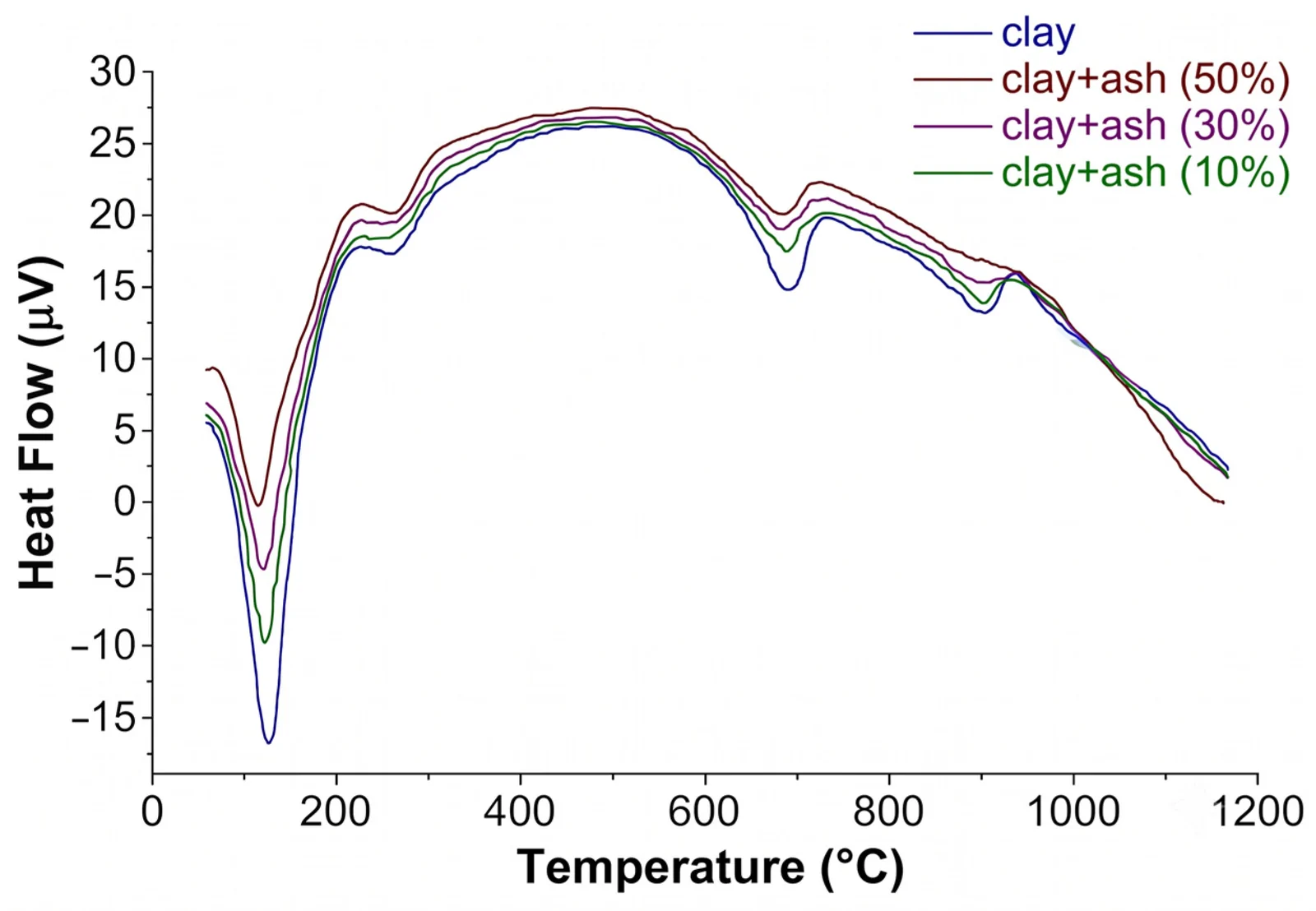
Differential thermal analysis (DTA) of the initial Saryozek clay and binary ash–clay compositions at a compaction pressure of 30 MPa.

A comparative analysis of the thermogravimetric and differential thermal curves (Figure 3 and Figure 4), combined with our XRD data, allowed for the establishment of the nature of thermal decomposition and phase evolution in the components and revealed a kinetic shift in the reactions under the influence of compaction pressure. In the low-temperature range (100–200 °C), the pure Saryozek clay exhibits a maximum mass loss (~8.0%), which is attributed to the removal of interlayer water from the montmorillonite. As the CHPP-2 fly ash concentration increases from 10% to 50%, the magnitude of this loss expectedly decreases due to the batch leaning effect. Meanwhile, increasing the pressure from 20 MPa to 30 MPa delays the removal of residual interlayer water up to 240–250 °C due to forced capillary contraction and an increase in the diffusion resistance of the over-compacted green body.

The most pronounced effect of the molding pressure is manifested in the mid-temperature region of dehydroxylation of the montmorillonite matrix. At a pressure of 20 MPa (Figure 3a), the removal of structural OH^−^ groups proceeds dynamically within the range of 480–560 °C, whereas at 30 MPa (Figure 3b), this process shifts to the range of 580–720 °C due to an increase in the partial vapor pressure within the dense pores. Although the dehydroxylation interval of montmorillonite under a compaction pressure of 30 MPa extends up to 720 °C due to increased internal vapor pressure, the strategic isothermal hold at 600 °C is specifically designated for the safe and complete burnout of the 5.0% carbonaceous unburned residue and initial moisture removal prior to the main structural dehydroxylation phase. Concurrently, in the range of 450–850 °C, the curve of the individual fly ash exhibits a smooth mass loss (~5.2%), corresponding to the burnout of 5.0% residual coal carbon. In the high-temperature region (950–1050 °C), the rigid mullite–quartz framework of the fly ash acts as a reinforcing skeleton, preventing the pyroplastic deformation of the clay [30]. At a pressure of 30 MPa and a temperature of 1050 °C, maximum synergism is achieved: the softened matrix effectively encapsulates the densely packed ash particles, providing an extreme increase in strength up to 38.4 MPa. The identified thermal shift in dehydroxylation justifies the necessity of holding the temperature at 600 °C for the safe removal of gases prior to the onset of intensive liquid-phase sintering. Furthermore, during the joint annealing of the montmorillonite–ash composite at temperatures exceeding 1000 °C, the initial layered structure of montmorillonite undergoes thermal destruction, and the reactive aluminosilicate components of the ash and clay engage in solid-state interactions, resulting in the crystallization of mullite and spinel phases as it was shown earlier by Valášková et al. [31].

For a more detailed identification of the thermodynamic effects and phase formation occurring without mass loss in the high-temperature sintering region of the composites, the differential thermal analysis (DTA) curves were analyzed at the optimal compaction pressure of 30 MPa (Figure 4).

On the obtained DTA curves, a deep endothermic peak is clearly recorded in the range of 100–200 °C with a pronounced minimum at 125 °C, which energetically confirms the intensive energy expenditure for the desorption and removal of the interlayer water of montmorillonite. The shift in the minimum of the second endothermic effect of matrix dehydroxylation to the region of 690 °C visually verifies the recorded kinetic TG shift in the decomposition of hydrosilicates under elevated pressure. In the high-temperature section (850–950 °C), a distinct exothermic peak with a maximum at 925 °C is detected, the intensity of which expectedly decreases as the clay component is replaced by the ash filler. This exo-effect is responsible for the final destruction of the anhydrous montmorillonite framework and the solid-phase crystallization of new crystalline phases (aluminosilicate spinel and metastable cristobalite) [32].

Upon final heating to 1050 °C, the rigid mullite–quartz framework of the CHPP-2 fly ash (mullite—45.6%, quartz—36.3%) acts as a reinforcing skeleton, preventing the pyroplastic deformation of the clay. The melt of the destructured clay effectively encapsulates the densely packed ash particles, blocking the porosity from the burned-out carbon and providing an extreme increase in the composite strength up to 38.4 MPa (for 10% ash). Thus, the comprehensive thermal data scientifically substantiate the selected firing regime with a technological holding step at around 600 °C for the safe removal of constitutional gases and unburned residue prior to the onset of intensive liquid-phase sintering of the green body.

**Figure 5:**
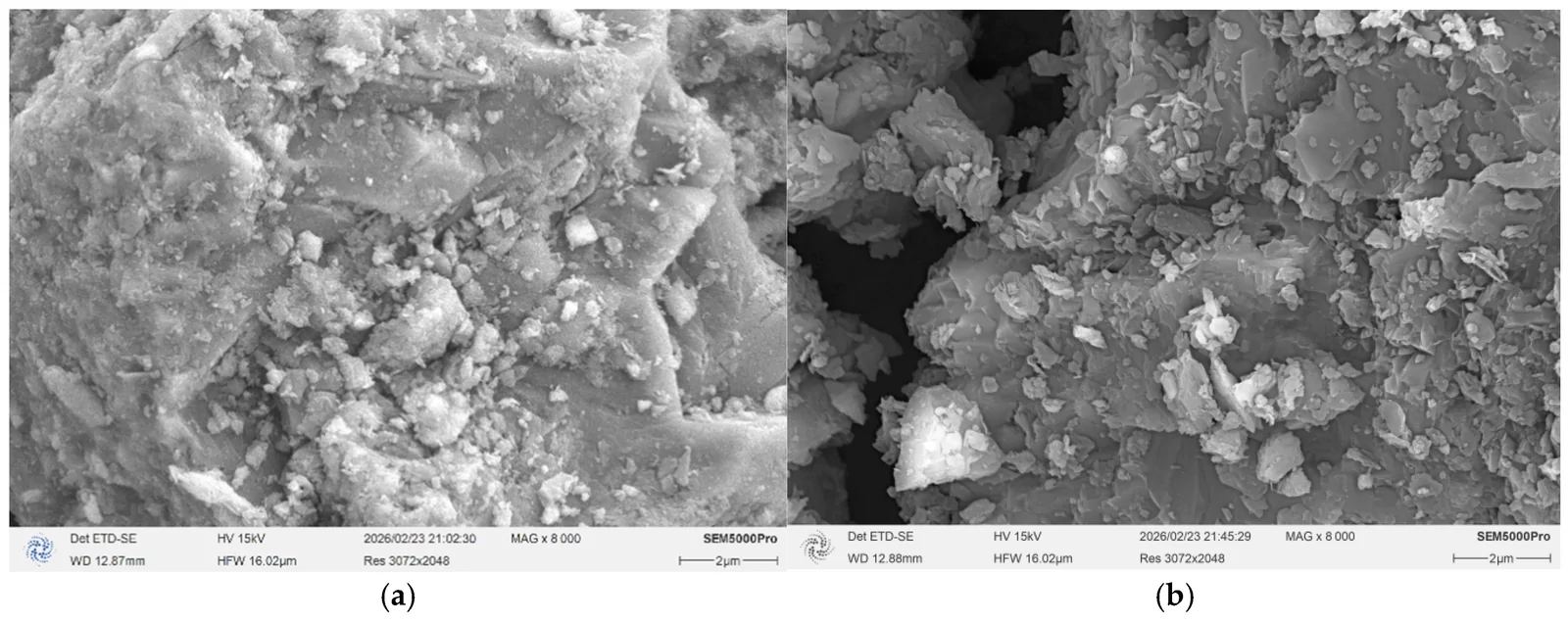
SEM micrographs of chipped clay composites after firing at 1050 °C (magnification ×8.000): (**a**) optimal composition (10% ash), demonstrating monolithic cementation of frame particles; (**b**) high-ash composition (50% ash), demonstrating a porous structure due to intense gas emission.

For the direct verification of the physicochemical processes of liquid-phase sintering and the mechanisms of pore formation, an SEM investigation of the fractured surfaces of the fired samples was conducted (Figure 5).

SEM of the sample with the optimal fly ash content of 10% (Figure 5a) records the formation of a dense, highly consolidated monolithic structure. Forced capillary contraction under the action of a 30 MPa compaction pressure ensured a maximum interfacial contact area. The locally formed silicate melt of the destructured Saryozek montmorillonite clay completely encapsulated the rigid, angular microparticles of mullite and quartz from the CHPP-2 fly ash, firmly cementing them into a matrix. At the macro level, this governs the extreme compressive strength value of 38.4 MPa.

Conversely, the fractured surface of the high-ash composite (50% ash, Figure 5b) reveals the development of open-pore defects with diameters of 1.5–5 μm. This morphology serves as a direct consequence of the intense gas evolution processes recorded by TG/DTA during montmorillonite dehydroxylation and the burnout of the 5.0% carbonaceous unburned residue of the ash. The gas phase “bloats” the pyroplastic melt of the matrix, while a pronounced deficit of the plastic binding phase (with the clay fraction reduced to 50%) prevents the healing of these voids. This leads to the thinning of the interpore walls and an expected drop in the material strength [33].

Thus, a comparative analysis of the fracture topology clearly confirms that a molding pressure of 30 MPa provides the necessary spatial convergence of the components, guaranteeing the monolithic cementation of the mullite–quartz framework by the silicate melt. However, the discovered synergistic effect is completely exhausted at ash concentrations exceeding 20–30%, where the deficit of the plastic binder, combined with intense destructive gas evolution, shifts the system into a regime of brittle structure formation. The established microstructural regularities are in strict concordance with the TG/DTA and physicomechanical testing results, fully revealing the physicochemical nature of the strength optimum of the developed composite ceramics.

Limitations and Future Perspectives. This study has several limitations that define directions for future research. First, the experimental program was implemented exclusively on a laboratory scale using precision, small-scale equipment. Under actual manufacturing conditions at an industrial plant, the heat and mass transfer profiles, as well as the gas evolution kinetics of the carbonaceous unburned residue, may undergo alterations due to the “scale-up effect.” This will require additional adjustment of the time intervals and heating rates using semi-industrial or pilot-scale installations.

Second, the evaluation of the performance viability of the developed aluminosilicate composites was limited at this stage to determining their basic physicomechanical properties. To fully validate the durability of the material under real operating conditions, long-term testing for frost resistance and abrasion resistance is required, along with an assessment of environmental aging and the chemical resistance of the formed matrix in aggressive media. The dense structural topology and the encapsulation of the refractory mullite–quartz framework by the silicate melt, revealed by SEM, provide reliable prerequisites for high operational performance, the detailed investigation of which will form the basis of the next stage of research.

## 3. Materials and Methods

### 3.1. Source Materials

The work uses natural clay materials of two different structural and mineralogical types as the main plastic matrix components:

1. The clay of the Saryozek deposit (Almaty region, Kazakhstan).
2. The clay of the Alekseevskaya deposit (Akmola region, Kazakhstan).

The fly ash from the CHPP-2 in Almaty, obtained as a result of high-temperature combustion of coal from the Ekibastuz basin, was used as a man-made filler and leaching component. The choice of this component is due to its aluminosilicate composition and dispersion, potentially comparable to traditional mineral thickeners.

### 3.2. Methods of Analysis and Physico-Mechanical Testing

The granulometric (sieve) analysis was performed by mechanical sieving of dispersed particles by size classes using a set of standard laboratory sieves.

The chemical (oxide) composition of the initial industrial and natural materials was determined by X-ray fluorescence analysis (XRF). The research was carried out using a high-precision wave-dispersive X-ray fluorescence spectrometer of the Axios series (manufactured by PANalytical, Almelo, The Netherlands), which allows precision quantitative determination of the content of basic rock-forming and glass-forming oxides.

Micromorphological analysis (visualization of the geometric shape of particles, the nature of their spatial organization and aggregation) was carried out using SEM on a SEM5000Pro microscope (manufactured by CIQTEK Co., Ltd., Hefei, China). The survey was conducted in the secondary electron mode at an accelerating voltage of 15 kV.

X-ray Diffraction analysis (XRD) was performed on an automated diffractometer DRON-4 using the powder diffractometry method (radiation was recorded in the angular range θ from 10° to 70°). Qualitative identification of crystalline phases and quantitative calculation of mineral composition were performed using specialized EVA (Version 6) and PCPDFWIN software (Version 2.1) using the international diffractometric database PDF-2. The following Standard ICDD PDF Card Numbers were used: Quartz 00-046-1045; Kaolinite 00-005-0143; Montmorillonite 00-013-0135; Mullite 00-015-0776; Illite 00-026-0911; Sillimanite 00-038-0471. Quantitative phase analysis of the studied materials (presented in Table 3, Table 4 and Table 5) was performed using the reference intensity ratio method based on the integrated intensities of characteristic diffraction peaks.

The plastic properties of clays were evaluated according to the ASTM standard method with experimental determination of the Liquid limit (W_L_) according to the Vasiliev cone, the Plastic limit (W_P_) by the thread-rolling method, and subsequent calculation of the plasticity index (I_P_ = W_L_ − W_P_). The physico-mechanical characteristics of the fired silicate shard (compressive strength, water absorption, fire shrinkage) were determined according to standard test methods for structural wall materials [34,35]. The compressive strength was calculated as the average value based on the test results of a series of samples on a hydraulic press.

### 3.3. Technology for Obtaining Composite Samples

The synthesis of composite ceramic samples was carried out by semi-dry pressing. On the basis of the binary system “clay Saryozek—ash CHPP-2”, molding charges were prepared with a strictly dosed content of the ash component: 10, 20, 30 and 50 wt.%. The dry components were subjected to mechanical homogenization, after which they were sealed with water. The molding humidity for the ash-free control sample was 10%, and for the ash-containing compositions it increased to 12% due to increased hygroscopicity and the developed specific surface area of the ash.

The cylindrical samples were molded in steel molds on a hydraulic press in the specific pressure range of 20 or 30 MPa in order to optimize the pressing conditions. After pressing, the samples were air-dried at room temperature for 24 h to remove free moisture, after which they were placed in a muffle furnace for high-temperature heat treatment.

The samples were fired at a maximum temperature of 950–1050 °C according to an optimized schedule:

1. The rate of temperature rise in the range of 20–200 °C was 2 °C/min (to prevent cracking during intensive removal of residual moisture).
2. In the range of 200–800 °C, the heating rate increased to 6 °C/min.
3. At a temperature of 600 °C, an isothermal exposure lasting 20 min was performed to ensure complete dehydration of clay minerals and combustion (burnout) of residual unburned carbon.
4. The final rise to the target temperature of 950–1050 °C was carried out at a rate of 1.5–5 °C/min, followed by a final isothermal exposure for 2.5 h.

The samples were cooled together with a muffle furnace to room temperature, after which they were subjected to mechanical tests for compressive strength.

### 3.4. Statistical Processing of Results

To ensure high reliability and reproducibility of the results of physico-mechanical tests (compressive strength, water absorption, fire shrinkage), each experimental parameter was determined as an arithmetic mean based on the test results of a series of at least 3 duplicate samples. Statistical processing of the obtained data sets was performed with the calculation of the standard deviation and confidence intervals at the significance level α = 0.05. The calculations were performed using the OriginPro 2024 software package.

## 4. Conclusions

Based on a comprehensive study of the physicochemical and processing properties of the raw materials, the choice of highly plastic Saryozek montmorillonite clay (I_P_ = 23.21%) as the base binding matrix has been scientifically substantiated. Unlike the polymineral clay from the Alekseevskaya deposit, the Saryozek clay possesses higher disparity and a labile crystal lattice, which allows it to effectively compensate for the non-plastic, rigid, clastic geometry of the mullite-quartz fly ash from Almaty CHPP-2 across the entire concentration range. A comparative thermal analysis (TG/DTA) established the fundamental regularities of firing and revealed a pronounced kinetic shift in the reactions under the influence of mechanical compaction of the developed ash–clay batches. Increasing the semi-dry molding pressure from 20 to 30 MPa delays the removal of interlayer water and shifts the dehydroxylation interval of the aluminosilicate matrix toward higher temperatures (580–720 °C with an endo-effect minimum at 690 °C) due to the increased diffusion resistance of the over-compacted green body. This shift scientifically argues for the introduction of an isothermal holding step at a temperature of 600 °C, which is necessary for the safe evacuation of gases prior to the onset of active liquid-phase sintering.

It was established that the optimal processing parameters for the synthesis of the construction composite ceramics are a molding pressure of 30 MPa, a dosed introduction of the ash component in an amount of 10–20 wt.%, and a maximum firing temperature of 1050 °C. Elevating the compaction force to 30 MPa provides forced capillary contraction and a synergistic increase in the strength of the ash–clay systems across the entire polythermal interval. The ultimate compressive strength value was recorded for the composite with 10% ash at 1050 °C, reaching 38.4 MPa at a controlled level of water absorption (14.92%) and firing shrinkage (6.15%). Exceeding the temperature threshold of 1050 °C is impractical, as it activates the processes of pyroplastic softening of the silicate melt and initiates destructive gas bloating of the products.

The structural mechanism of sintering of the binary aluminosilicate systems at the extreme points of strength characteristics was fully verified using SEM. In the optimal formulations (10% ash) under a pressure of 30 MPa, the locally formed liquid phase of the destructured montmorillonite completely encapsulates and monolithically cements the rigid, framework microparticles of mullite and quartz from the ash, forming a green body. Conversely, with excessive loading of the batch with the industrial waste (50% ash), the development of a porous cellular topology with open pores is recorded. This morphology is attributed to the evolution of water vapor and CO_2_ from the burnout of the 5.0% carbonaceous unburned residue of the ash under conditions of a pronounced deficit of the plastic clay binder, which is independently and comprehensively proven by the shifts in energetic effects on the TG and DTA curves. The evolved gas phase leads to the thinning of the interpore walls and an expected drop in the strength of the macrostructure of the high-ash composites.

The developed optimal molding (30 MPa) and firing parameters (1050 °C with an isothermal holding step at 600 °C) lay a scientifically substantiated foundation for reducing defects during heat treatment. Nonetheless, given the exclusively laboratory scale of the conducted tests, the transition to real high-tonnage production will require additional verification of the processing parameters in pilot-scale or semi-industrial tunnel kilns to account for the scale-up factor, heat and mass transfer characteristics, and dynamic loads on the kiln cars.

## Figures and Tables

**Table 1:** Granulometric composition of the studied samples.

Samples	Particle Size, %		
	<0.056 mm	On Sieve 0.056 mm	On Sieve 0.63 mm
Fly Ash of CHPP-2	29.5 ± 0.4	35.3 ± 0.7	34.2 ± 0.5
Alekseevskaya Clay	38.1 ± 0.6	49.4 ± 0.7	12.5 ± 0.6
Saryozek Clay	46.2 ± 0.8	33.6 ± 0.6	20.2 ± 0.4

**Table 2:** Chemical composition of the two types of clay and ash of CHPP-2 studied.

The Content of Oxides, %											
Samples	SiO_2_	Al_2_O_3_	CaO	Fe_2_O_3_	MgO	TiO_2_	SO_3_	K_2_O	Na_2_O	C	R_2_O
Fly Ash of CHPP-2	58.8	21.26	2.83	6.6	0.35	1.9	0.72	0.38	0.36	5.0	1.8
Alekseevskaya Clay	58.9	24.02	0.66	1.05	0.22	0.45	-	3.5	2.08	-	8.02
Saryozek Clay	64.78	22.36	0.29	1.29	0.53	1.25	-	2.34	-	-	6.78

**Table 3:** Quantitative phase composition of CHPP-2 ash.

Mineral Content, %								
The raw material	Glass phase	Quartz	Mullit	Calcite	Sillimanite	Albit	Rutile	Magnesite
Fly Ash of CHPP-2	4.6 ± 0.3	36.3 ± 0.3	45.6 ± 0.4	3.5 ± 0.2	2.4 ± 0.2	5.8 ± 0.4	0.9 ± 0.2	0.9 ± 0.2

**Table 4:** Quantitative phase composition of Saryozek clay.

Mineral Content, %						
Hydrosilicate	Quartz	Gypsum	Feldspar	Kaolinite	Montmorillonite	Iron oxides
9.5 ± 0.7	27.8 ± 0.7	3.2 ± 0.3	7.8 ± 0.4	5.1 ± 0.3	45.4 ± 0.8	4.2 ± 0.2

**Table 5:** Quantitative phase composition of Alekseevskaya clay.

Mineral Content, %					
Hydrosilicate	Quartz	Gypsum	Feldspar	Kaolinite	Iron oxides
15.1 ± 0.7	30.2 ± 0.8	3.5 ± 0.3	2.8 ± 0.2	43.2 ± 0.8	4.2 ± 0.2

**Table 6:** Plasticity of clay raw materials.

Clay	Liquid Limit, %	Plastic Limit, %	Plasticity Index
Saryozek Clay	59.03 ± 0.33	35.82 ± 0.25	23.21 ± 0.22
Alekseevskaya Clay	35.85 ± 0.28	21.45 ± 0.31	14.40 ± 0.18

**Table 7:** Physical and mechanical characteristics of clays.

Sample	Firing Temperature, °C	Water Absorption, %	Total Shrinkage, %	Compressive Strength, MPa
Alekseevskaya clay (Kaolinitic type)	950	15.9 ± 1.2	3.15 ± 0.3	16.8 ± 0.7
	1000	14.6 ± 1.3	3.64 ± 0.3	19.87 ± 0.9
	1050	12.9 ± 1.1	4.15 ± 0.4	24.9 ± 1.2
	1100	9.4 ± 0.9	5.68 ± 0.6	31.2 ± 1.5
	1150	8.2 ± 0.8	8.1 ± 0.6	33.0 ± 1.3
	1200	4.5 ± 0.5	6.7 ± 0.5	35.1 ± 1.2
Saryozek clay (Montmorillonitic type)	950	18.2 ± 1.5	3.15 ± 0.3	12.4 ± 1.1
	1000	14.1 ± 1.3	5.82 ± 0.6	18.6 ± 1.4
	1050	9.8 ± 1.1	8.40 ± 0.7	25.3 ± 1.4
	1100	6.2 ± 0.5	10.12 ± 0.9	28.9 ± 1.2
	1150	5.5 ± 0.4	9.40 ± 0.9	24.1 ± 1.3
	1200	—	—	Pyroplastic deformation

**Table 8:** Physical and mechanical properties of the investigated CHPP-2 fly ash.

Parameter Description	Unit	Value
Appearance		Light gray powder
Bulk density in as-received state	kg/m^3^	900 ± 50
True density of grains	g/cm^3^	1.98
Specific surface area	m^2^/kg	260–280
Melting temperature	°C	1430
Softening point	°C	1300

**Table 9:** Classification criteria of raw materials.

Module	CHPP-2 Fly Ash	Alekseevskaya Clay	Saryozek Clay
BM	0.062	0.120	0.009
AM	2.760	2.450	2.890
SM	2.110	2.350	2.740
Qc	0.360	0.407	0.345

**Table 10:** Chemical composition of the batch mixtures.

Mixture Composition, %		Oxide Content, %										
Saryozek clay	CHPP-2 fly ash	SiO_2_	Al_2_O_3_	Fe_2_O_3_	MgO	TiO_2_	SO_3_	K_2_O	Na_2_O	C	R_2_O	CaO
100	-	64.78	22.36	1.29	0.53	1.25	-	2.34	-	-	6.48	0.29
90	10	61.48	22.25	1.82	0.50	1.31	0.07	2.02	0.04	0.50	6.28	0.54
80	20	61.18	22.13	2.35	0.49	1.38	0.14	1.91	0.07	1.04	5.78	0.79
70	30	60.83	22.03	2.88	0.48	1.45	0.22	1.75	0.11	1.50	5.08	1.05
50	50	60.60	21.81	3.95	0.42	1.58	0.36	0.72	0.18	2.50	4.11	1.56

**Table 11:** Experimental design options and physical and mechanical characteristics of the samples after firing. Molding pressure 20 MPa.

CHPP-2 Fly Ash Content, wt.%	Firing Temperature °C	Firing Shrinkage, %	Water Absorption, %	Compressive Strength, MPa
0	950	2.7 ± 0.3	14.5 ± 0.8	23.3 ± 1.3
	1000	4.7 ± 0.3	13.1 ± 0.7	29.8 ± 1.4
	1050	8.4 ± 0.6	10.2 ± 0.6	24.1 ± 1.3
10	950	2.8 ± 0.2	22.3 ± 0.8	20.9 ± 1.2
	1000	3.65 ± 0.4	21.5 ± 0.7	21.1 ± 1.4
	1050	6.5 ± 0.5	18.3 ± 0.8	23.9 ± 1.3
20	950	1.85 ± 0.2	22.5 ± 0.7	12.4 ± 1.1
	1000	2.5 ± 0.3	18.3 ± 0.7	18.9 ± 1.1
	1050	3.7 ± 0.3	18.3 ± 0.6	21.4 ± 1.2
30	950	1.2 ± 0.2	22.3 ± 0.7	14.1 ± 1.1
	1000	1.5 ± 0.2	25.4 ± 0.8	18.5 ± 1.3
	1050	2.3 ± 0.2	24.2 ± 0.6	22.4 ± 1.4
50	950	1.0 ± 0.1	26.7 ± 0.7	10.2 ± 0.9
	1000	1.2 ± 0.1	25.1 ± 0.8	11.7 ± 0.9
	1050	1.9 ± 0.1	22.6 ± 0.6	16.9 ± 1.1

**Table 12:** Experimental design options and physical and mechanical characteristics of the samples after firing. Molding pressure 30 MPa.

CHPP-2 Fly Ash Content, wt.%	Firing Temperature °C	Firing Shrinkage, %	Water Absorption, %	Compressive Strength, MPa
0	950	2.75 ± 0.3	14.5 ± 0.8	33.15 ± 1.3
	1000	4.81 ± 0.4	13.0 ± 0.9	36.9 ± 1.5
	1050	8.6 ± 0.6	10.4 ± 0.9	32.0 ± 1.5
10	950	2.8 ± 0.3	18.05 ± 1.1	31.8 ± 1.4
	1000	3.65 ± 0.3	17.25 ± 0.9	34.9 ± 1.5
	1050	6.15 ± 0.4	14.92 ± 0.8	38.4 ± 1.3
20	950	1.85 ± 0.2	22.3 ± 0.9	14.9 ± 1.3
	1000	2.5 ± 0.2	21.5 ± 1.1	20.0 ± 1.4
	1050	3.7 ± 0.3	18.1 ± 0.8	26.3 ± 1.4
30	950	1.2 ± 0.1	25.4 ± 1.1	13.8 ± 1.3
	1000	2.5 ± 0.2	24.2 ± 1.1	17.3 ± 1.3
	1050	2.1 ± 0.2	21.8 ± 1.1	21.4 ± 1.4
50	950	1.0 ± 0.1	26.75 ± 1.2	8.1 ± 1.0
	1000	1.2 ± 0.1	25.2 ± 0.9	9.1 ± 0.7
	1050	1.9 ± 0.1	22.6 ± 1.1	17.9 ± 1.0

## Data Availability

The original contributions presented in this study are included in the article. Further inquiries can be directed to the corresponding author.

## Abbreviations

The following abbreviations are used in this manuscript:

CHPP	Combined Heat and Power Plant
XRD	X-ray Diffraction
XRF	X-ray Fluorescence
TG/DTA	Thermogravimetry/Differential Thermal Analysis
SEM	Scanning Electron Microscope
I_P_, W_L_, W_P_	The Atterberg limits: Plasticity Index, Liquid Limit, Plastic Limit
